# Health literacy and diabetes information preferences among Chinese immigrants: An Australian cross‐sectional study

**DOI:** 10.1002/nop2.70029

**Published:** 2024-09-14

**Authors:** Shanshan Lin, Danielle Muscat, Julie Ayre

**Affiliations:** ^1^ Diabetes Course Director, School of Public Health University of Technology Sydney Sydney New South Wales Australia; ^2^ Sydney Health Literacy Lab, School of Public Health, Faculty of Medicine and Health The University of Sydney Sydney New South Wales Australia

**Keywords:** Chinese immigrants, diabetes, health literacy, patient education materials

## Abstract

**Aim:**

Explore Australian‐Chinese immigrants' health literacy and preferences and engagement with translated diabetes self‐management patient education materials.

**Design:**

The cross‐sectional survey was conducted with Australian‐Chinese immigrants at risk or with type 2 diabetes recruited via health services, and diabetes and community organisations.

**Methods:**

The survey had three parts: (1) diabetes screening; (2) sociodemographic information, clinical characteristics and preferences for translated materials; and (3) Functional, Communicative and Critical Health Literacy (FCCHL) Scale.

**Results:**

Of 381 participants, 54.3% reported diabetes (*n* = 207), the remainder pre‐diabetes or at risk (45.7%, *n* = 174); 34.1% male; mean age 64.1 years. Average total health literacy (FCCHL) scores were 35.3/56 (SD = 8.7). Participants with greater English proficiency reported higher health literacy (*p* < 0.001). This pattern also existed for functional (*p* < 0.001), communicative (*p* = 0.007) and critical (*p* = 0.041) health literacy subdomains. Health literacy scores did not differ significantly based on years of residence in Australia (all *p* > 0.05). Although the majority of participants (75.6%, *N* = 288) were willing to receive translated diabetes information, only a small proportion (19.7%, *N* = 75) reporting receiving such materials.

**Conclusion:**

There is a clear need for co‐designed diabetes patient education materials that meet the needs and adequately reach Australian‐Chinese immigrants. In particular, these materials must support people with limited English‐language proficiency.

**Implications for Nursing Practice:**

This study highlights important considerations for nurses seeking to improve diabetes care for Chinese immigrants when incorporating patient education materials as part of their nursing education.

## 1 BACKGROUND

Many culturally and linguistically diverse (CALD) communities in Western countries experience greater prevalence of type 2 diabetes compared to the general population. In Australia, the estimated prevalence for one of the largest language groups, the Chinese‐speaking community, is as high as 17%, compared to 7.4% in the general population (Colagiuri et al., 2007). Whilst this higher prevalence can be partially attributed to genetic and socio‐economic factors, Chinese immigrants also face substantial language barriers that can bring additional complexity in providing health care (Zhang et al., 2020). To illustrate, in 2016 an estimated 40% of Chinese‐speaking Australians aged 50–59 years reported speaking English ‘not well’ or ‘not at all’ (ABS, 2018).

Health literacy is also relevant to diabetes prevention and care. Health literacy enables a person to learn about and engage in diabetes self‐management for example, take medicines safely, make effective dietary choices and take actions based on blood glucose readings. Low health literacy is associated with poorer diabetes knowledge and glycaemic management, and higher diabetes distress (Marciano et al., 2019). Health information and services also contribute to health literacy. Diabetes self‐management education materials (hereafter referred to as ‘materials’), delivered via classes or digital technologies are crucial to providing clinically meaningful improvements in diabetes outcomes (Hermanns et al., 2020). However, qualitative research suggests that in practice, these materials fall short of the needs of Chinese immigrants and can be difficult to find (Choi et al., 2018). These shortcomings are likely to perpetuate health inequality for this group.

In Australia, much of the diabetes prevention and self‐management education across hospitals, communities and primary care is delivered by Credentialled Diabetes Educators. Although Credentialled Diabetes Educators include a range or professions, nurses have and continue to be the primary profession of this workforce (King et al., 2017; Murfet et al., 2022). Given their central role and rich experience supporting people with diabetes from Chinese immigrant communities, nurses are therefore in a prime position to improve education materials so that they better meet community needs. The current study aimed to support nurses in this endeavour by quantitatively exploring:

- The relationship between health literacy and proficiency in English and Chinese language and other relevant demographic variables, for Australian Chinese immigrants with or at risk of type 2 diabetes;
- Australian Chinese immigrants' engagement, attitudes and preferences for diabetes self‐management education materials.

## 2 METHODS

### 2.1 Design

Cross‐sectional community survey data were collected between March and December, 2018. This study was approved by the University of Sydney Human Research Ethics Committee (Ethics no: 2018/063).

### 2.2 Recruitment

Eligible participants self‐reported a diagnosis of type 2 diabetes or at risk (prediabetes), or scored in the high‐risk category of AUSDRISK, an Australian validated screening tool for identifying people at high risk of developing type 2 diabetes in the next 5 years (Chen et al., 2010). Participants also had to be over 18 years, born overseas and living in Australia, and able to read Simplified Chinese.

Participants were recruited through partner organisations (e.g. NSW Multicultural Health, State branches of Diabetes Australia and key Chinese community organisations). Participants could also encourage others to complete the survey.

### 2.3 Survey

After consenting, participants completed items about demographics, language proficiency for written English, Simplified Chinese and Traditional Chinese (assessed on a three‐point scale (‘poor,’ ‘ok,’ ‘good’)), and clinical characteristics. Participants were then asked about materials they had received in China and Australia, willingness to engage with in‐language materials, and preferences for materials from China versus Australia. Written free‐text responses were captured in Chinese. Lastly, participants completed the simplified Chinese version of the Functional, Communicative and Critical Health Literacy scale (FCCHL) (Ishikawa et al., 2008, Zhang et al., 2018), with minor amendments to ensure it was suitable for the Australian context. This is a validated 14‐item self‐reported measure of health literacy, that estimates total health literacy (*α* = 0.88) and three subdomains. Five items relate to functional health literacy (*α* = 0.93) and communicative health literacy (*α* = 0.93). Four relate to critical health literacy (*α* = 0.92). Participants responded using a four‐point Likert‐type scale (‘never’, ‘rarely’, ‘sometimes’ and ‘often’). Total scores and sub‐totals for each subdomain were calculated by summing responses. Higher scores indicate higher health literacy.

Free‐text comments were translated into English by SL (see Appendix for all details). All comments were grouped into themes by SL and DM independently for content analysis. Discrepancies were resolved through discussion with all authors. Quantitative data and content analysis themes were then analysed descriptively. Differences in total and subdomain FCCHL scores across variables of interest were compared using one‐way ANOVAs.

## 3 RESULTS

### 3.1 Demographic characteristics and health literacy

Of 381 participants, 54.3% (*N* = 207) reported having diabetes; the remainder had been diagnosed with prediabetes or were considered at high risk using the AUSDRISK survey (Table 1). Of those diagnosed with diabetes, half (47.8%, *N* = 99) reported that they had lived with the condition for more than 5 years. The average age was 64.1 years (SD = 14.2), and two thirds (63.8%; *N* = 243) were female. The majority (79.3%, *N* = 302) were born in China and had lived in Australia for more than 10 years (61.2%, *N* = 233). Two thirds (64.6%, *N* = 246) reported less than a university level education.

**TABLE 1:** Clinical and demographic characteristics of the analysis sample (*N* = 381).

	N	% or mean (SD)	Total FCCHL (M (SD))^a^
*Diabetes status* ^b^			
Diabetes	207	54.3	41.7 (9.1)
Prediabetes or high risk of diabetes	174	45.7	38.5 (8.0)
*Duration of diabetes* ^c^			
Less than 12 months	17	8.2	43.3 (7.8)
1–5 years	59	28.5	41.9 (9.1)
6–10 years	43	20.8	43.3 (9.2)
More than 10 years	56	27.1	41.2 (8.7)
Unsure	32	15.5	38.2 (7.6)
Age (years; M, SD)	362	64.1 (14.2)	40.6 (8.6)
*Gender*			
Male	130	34.1	40.5 (8.6)
Female	243	63.8	40.1 (8.8)
Not stated	8	2.1	
*State of residence*			
Australian Capital Territory	20	5.2	37.4 (9.8)
New South Wales	303	79.5	39.9 (8.5)
Queensland	20	5.2	46.6 (8.4)
South Australia	12	3.1	40.3 (9.5)
Victoria	17	4.5	44.9 (8.2)
Not stated	9	2.4	
*Country of birth*			
China	302	79.3	40.0 (8.5)
Hong Kong/Macau	45	11.8	43.4 (8.8)
Taiwan	4	1.0	44.0 (11.3)
Vietnam, Malaysia or Singapore	16	4.2	37.3 (11.1)
Other	7	1.8	35.7 (5.3)
*Not stated*	7	1.8	
*Years living in Australia*			
Less than 12 months	23	6.0	39.5 (7.5)
1–5 years	67	17.6	39.0 (8.0)
6–10 years	56	14.7	41.8 (7.5)
More than 10 years	233	61.2	40.4 (9.2)
Not stated	2	0.5	
*Highest level of education*			
Less than university	246	64.6	38.8 (8.3)
University	128	33.6	43.3 (8.8)
Not stated	7	1.8	
*Proficiency in simplified Chinese*			
Low	16	4.2	33.3 (8.5)
Medium	129	33.9	38.6 (8.3)
High	211	55.4	41.8 (8.7)
Not stated	25	6.6	
*Proficiency in traditional Chinese*			
Low	52	13.6	35.9 (9.5)
Medium	145	38.1	40.4 (9.0)
High	84	22.0	43.0 (8.8)
Not stated	100	26.2	
*Proficiency in English*			
Low	170	44.6	37.7 (8.5)
Medium	82	21.5	42.7 (8.4)
High	48	12.6	44.2 (9.3)
Not stated	2	0.5	

^a^ Some participants did not complete the FCCHL. These participants are excluded from calculation of M and SD for total FCCHL scores.

^b^ 25 participants did not state diabetes status but scored in the high‐risk category of the AUSDRISK screening tool.

^c^ Three participants with diabetes did not report how long they had the condition.

Participants reported higher language proficiency for Simplified Chinese (55.4% reporting ‘high’ proficiency; *N* = 211), compared to Traditional Chinese (22.0% reporting ‘high’ proficiency, *N* = 84). English language proficiency was generally poor (only 12.6% reporting ‘high’ proficiency, *N* = 48). Proficiency in Simplified and Traditional Chinese were positively associated (*χ* ^2^(4) = 28.1, *p* < 0.001); proficiency in English was associated with proficiency in Simplified Chinese (*χ* ^2^(4) = 11.0, *p* = 0.027) and Traditional Chinese (*χ* ^2^(4) = 53.9, *p* < 0.001).

Average total health literacy score was 35.3 out of a possible 56 (SD = 8.7). Average scores were 18.4 out of 25 (SD = 4.9) for functional health literacy, 13.4 out of 25 (SD = 4.5) for communicative health literacy, and 9.4 out of 20 (SD = 3.8) for critical health literacy. Communicative and critical health literacy scores were positively associated (*r* = 0.66, *p* < 0.001).

On average, participants with stronger English proficiency had higher total (*p* < 0.001), functional (*p* < 0.001), communicative (*p* = 0.007), and critical health literacy scores (*p* = 0.041). The same pattern was observed for Chinese‐language proficiency with the exception of functional health literacy (all *p* < 0.02). Health literacy scores did not differ significantly by years living in Australia.

### 3.2 Diabetes education engagement, attitudes and preferences

One hundred and fifty‐three (40.2%) participants reported that they had engaged with Australian diabetes healthcare professionals (including diabetes specialists, diabetes educators and dietitians). Most reported that they had not received diabetes education in their home country (72.2%, *N* = 275).

One fifth of participants (19.7%, *n* = 75) had received Chinese‐language diabetes information from Diabetes Australia (or corresponding state/territory counterparts) and the National Diabetes Service Scheme, two of the leading peak bodies for diabetes in Australia. However, most (75.6%, *n* = 288) reported willingness to receive Australian materials in Chinese language, and preferred Simplified Chinese materials from Australia (63.5%, *n* = 242) rather than China (29.9%, *n* = 114).

The most common reason for preferring materials from Australia was that the information would be more practical (54.5%, *n* = 132) (Table A1). The most common reason for preferring materials from China was that the language and writing style would be more appropriate (54.4%, *n* = 62). Fifty‐seven participants gave further detail about their information preferences, all of whom had lived in Australia less than 5 years, summarised in Table 2.

**TABLE 2:** Themes from the free‐text comments about diabetes material preferences.

Themes	Description	Example quotes	No. of responses
1: Locally relevant information	Expressions about the needs and importance of local information, mainly due to the immigration or relocation	‘Good to have the local information’.	35.1% (*n* = 20)
2: Cultural and genetic predispositions	Concerns about the suitability of westernised food information and medical treatments for their own Chinese constitution	‘The genetics were different, so the [clinical] targets [for the diabetes management] were different’.	14.0% (*n* = 8)
3: Diet and Complementary and Alternative Medicine (CAM)	Dietary related concerns or comments, and expressions about the CAM	‘Had information about the Chinese medicine and practical examples’.	7.0% (*n* = 4)
4: Credibility/trust	Perceived high trust or higher quality of the published materials	‘The Australian information was more trustable’.	14.0% (*n* = 8)
5: Other	Any other comments that didn't meet any of above themes	‘I wanted both materials, so I could compare Australian and Chinese information’.	29.8% (*n* = 17)

## 4 DISCUSSION

In this sample of Chinese immigrants with type 2 diabetes or at risk in Australia, self‐reported health literacy was higher for people with greater proficiency in English and Simplified Chinese. Number of years spent living in Australia was not associated with health literacy. Despite high willingness to receive diabetes self‐management materials and a strong preference for in‐language materials from Australia, only a small proportion had actually received such materials from Australian organisations. Participants valued Australian‐based diabetes self‐management education materials because they were perceived as providing more relevant practical information and understandable design.

This study contributes to existing research on the relationship between health literacy and language proficiency. Although years of living in a country has been associated with higher health literacy among immigrants (Australian Bureau of Statistics, 2019), this relationship was not observed in our own work and others' (Ishikawa et al., 2008). Participants' strong interest in receiving diabetes information in our study is also consistent with qualitative research reporting that Australian Chinese immigrants want diabetes self‐management education materials that provide practical, local information, relevant dietary information and culturally adapted visual aids (Choi et al., 2018). More broadly, the study findings echo other Australian nursing research that has called for more inclusive, culturally sensitive and appropriate health educaton materials to better meet the needs of Chinese immigrant communities (Jin et al., 2020; Li et al., 2018; Zeng et al., 2023).

A key strength of this study is the large sample from communities that typically have low rates of participation in research. However, the self‐reported nature of the data may limit the validity of study findings. In particular, diabetes status was not medically verified. Although we recruited across several sites and community networks, convenience sampling is unlikely to be representative.

### 4.1 Implications for nursing practice

In Australia, nurses and midwives are the primary non‐medical professionals delivering diabetes education (King et al., 2017), and influential advocates and leaders for enhanced diabetes care (Murfet et al., 2022). This study highlights important considerations for nurses seeking to improve diabetes care for Chinese immigrants: (1) A person with poor language proficiency in English or Chinese may be more likely to have low health literacy; (2) Many patients who are Chinese‐Australian immigrants may not be aware of in‐language materials available from Australian diabetes organisations. Some may prefer materials developed in China or overseas; (3) Further work is needed to create in‐language health information that uses simple language and provides practical local information, developed in partnership with members of these communities.

## 5 CONCLUSION

Chinese Australian immigrants with type 2 diabetes or at risk with greater proficiency in English and Simplified Chinese reported higher health literacy. Participants reported limited access to translated diabetes self‐management education materials despite an overwhelming willingness to receive such information. This study supports the argument for co‐designing translated diabetes materials with immigrants, nurses and other diabetes educators, with an emphasis on practical information and easy‐to‐understand, culturally adapted visuals.

## AUTHOR CONTRIBUTIONS

All authors listed meet the criteria for the authorship as outlined in 4.1. Shanshan Lin conceived of the study. Shanshan Lin, Julie Ayre and Danielle Muscat were involved in study design, methods and analysis. Shanshan Lin, Julie Ayre and Danielle Muscat prepared the ethics application. Shanshan Lin conducted all recruitment, managed the data and drafted the manuscript, while Julie Ayre led the analysis of the data. All authors critically revised the manuscript, and read and approved the final manuscript.

## FUNDING INFORMATION

The author (SL) received Postgraduate Research Support Scheme from the University of Sydney to collect data for the study. Other authors (JA, DM) received no funding for this study.

## CONFLICT OF INTEREST STATEMENT

The authors declare that they have no competing interests.

## ETHICS STATEMENT

This study was approved by the University of Sydney Human Research Ethics Committee (2018/063).

## REPORTING CHECKLIST

The STROBE statement checklist was completed for this cross‐sectional study.

## Data Availability

The data that support the findings of this study are available on request from the corresponding author. The data are not publicly available due to privacy or ethical restrictions.

## APPENDIX A

**TABLE A1:** Reasons for preferring Chinese‐ or Australian‐based diabetes self‐management education materials (PEMs)^a^.

	*N* (%)	
	Preference for materials from China (*n*=114)	Preference for materials from Australia (*n*=242)
Language and writing style	62 (54.4)	67 (27.7)
Uses Chinese diabetes guidelines	50 (43.9)	12 (5.0)
Provides practical information	39 (34.2)	132 (54.5)
Easy‐to‐understand images and pictures	54 (47.4)	114 (47.1)
Other reasons	6 (5.3)	33 (13.6)

^a^ Four participants who indicated no preference are not included.
